# How Do We Recognize Emotion From Movement? Specific Motor Components Contribute to the Recognition of Each Emotion

**DOI:** 10.3389/fpsyg.2019.01389

**Published:** 2019-07-03

**Authors:** Ayelet Melzer, Tal Shafir, Rachelle Palnick Tsachor

**Affiliations:** ^1^Faculty of Social Welfare and Health Sciences, The Graduate School of Creative Arts Therapies, University of Haifa, Haifa, Israel; ^2^The Emili Sagol Creative Arts Therapies Research Center, University of Haifa, Haifa, Israel; ^3^School of Theater and Music, University of Illinois at Chicago, Chicago, IL, United States

**Keywords:** emotion recognition, Laban movement analysis, motor, emotion, movement, bodily emotional expressions, dance-movement therapy

## Abstract

Are there movement features that are recognized as expressing each basic emotion by most people, and what are they? In our previous study we identified sets of Laban movement components that, when moved, elicited the basic emotions of anger, sadness, fear, and happiness. Our current study aimed to investigate if movements composed from those sets would be recognized as expressing those emotions, regardless of any instruction to the mover to portray emotion. Our stimuli included 113 video-clips of five Certified Laban Movement Analysts (CMAs) moving combinations of two to four movement components from each set associated with only one emotion: happiness, sadness, fear, or anger. Each three second clip showed one CMA moving a single combination. The CMAs moved only the combination's required components. Sixty-two physically and mentally healthy men (*n* = 31) and women (*n* = 31), ages 19–48, watched the clips and rated the perceived emotion and its intensity. To confirm participants' ability to recognize emotions from movement and to compare our stimuli to existing validated emotional expression stimuli, participants rated 50 additional clips of bodily motor expressions of these same emotions validated by Atkinson et al. (2004). Results showed that for both stimuli types, all emotions were recognized far above chance level. Comparing recognition accuracy of the two clip types revealed better recognition of anger, fear, and neutral emotion from Atkinson's clips of actors expressing emotions, and similar levels of recognition accuracy for happiness and sadness. Further analysis was performed to determine the contribution of specific movement components to the recognition of the studied emotions. Our results indicated that these specific Laban motor components not only enhance feeling the associated emotions when moved, but also contribute to recognition of the associated emotions when being observed, even when the mover was not instructed to portray emotion, indicating that the presence of these movement components alone is sufficient for emotion recognition. This research-based knowledge regarding the relationship between Laban motor components and bodily emotional expressions can be used by dance-movement and drama therapists for better understanding of clients' emotional movements, for creating appropriate interventions, and for enhancing communication with other practitioners regarding bodily emotional expression.

## Introduction

In this study, we aimed to investigate emotion recognition from movement using the framework and language of Laban Movement Analysis (LMA). Pioneering empirical studies in the field of emotion recognition showed an association between specific facial expressions and emotions. Ekman (1982) described six biologically based emotional facial expressions, with which we are born and spontaneously understand in others, regardless of our cultural background. Continuing the idea of existing associations between facial expressions and emotions, it was argued that emotions are recognized not only from facial expressions, but also from whole body expressions and movements even in the absence of a facial expression, and that these associations should be studied as well (de Gelder, 2009, p. 3475–3484). Indeed, many studies have shown that participants successfully identified emotions from watching other people's bodily movements (without seeing facial expressions) far above what is expected by chance (De Meijer, 1989, p. 247–268; Wallbott, 1998, p. 879–896; Montepare et al., 1999, p. 133–152; Atkinson et al., 2004, p. 717–746; Crane and Gross, 2007, p. 95–101; Crane and Gross, 2013, p. 91–105). Moreover, Aviezer et al. (2012, p. 1225–1229) asked participants to rate the valence (positive or negative) and the intensity of the expressions of high-level tennis players at losing or winning situations. They chose these situations, because such situations tend to evoke strong affective responses. Their participants were asked to watch one of the three options: facial expressions alone, bodily expressions alone, or facial and bodily expressions together. As they predicted, participants failed to correctly rate “winning faces” as more positive and “losing faces” as more negative when they saw faces alone, but succeeded when they watched the body and face or only bodily expressions. Their results indicated that during peak emotional situations, facial expressions of negative and positive valence may overlap, and when this occurs, people use bodily expressions to infer the valence of the expressed emotion.

The associations between certain movements and specific emotions were also demonstrated in studies which found that having specific feelings elicited specific body movement patterns (Sawada et al., 2003, p. 697–708; Crane and Gross, 2007, p. 95–101; Michalak et al., 2009, p. 580–587; Roether et al., 2009, p. 15; Dael et al., 2012b, p. 1085; Crane and Gross, 2013, p. 91–105). For instance, Crane and Gross (2007, p. 95–101) elicited one of four feelings (angry, sad, content, and joy) in participants and then filmed them walking in a self-selected pace. They found that body movements were affected by the different emotions.

Looking at emotionally expressive movement from yet another perspective, some studies have found that body movements and postures elicit feelings and emotion-related behaviors (e.g., Strack et al., 1988, p. 768; Duclos and Laird, 2001, p. 27–56; Winters, 2008, p. 84–105; Carney et al., 2010, p. 1363–1368; Shafir et al., 2013, p. 219–227). Shafir et al. (2013, p. 219–227) investigated the effect of imagining and looking at others' emotional movement, and motor execution of emotion-related movements on mood, and found that executing body movements evoked their associated feelings. Imagining and looking at others' emotional bodily motor expressions also evoked the associated emotions in participants, but to a lesser degree. Carney et al. (2010, p. 1363–1368) investigated the influence of body postures on emotion. They asked participants to stay in either open/expansive “powerful” postures or closed, “weak/submissive” postures for 2 min. They found that participants who were positioned in the power poses not only felt more powerful and tended to take more risks, but also had higher testosterone levels and lower cortisol levels, while those positioned in the submissive postures tended to take less risks and had higher cortisol levels. Although a following study did not succeed to replicate the hormones related results (Ranehill et al., 2015, p. 653–656) and as a result some researchers questioned the effects of posture and movement on emotions (Simmons and Simonsohn, 2017, p. 687–693), a recent comprehensive review by Cuddy et al. (2018, p. 656–666) which analyzed this effect in 55 studies using a *p*-curve analysis, concluded that “Our *p*-curve analysis of emotion- and affect-related outcomes yielded robust evidence that postural feedback influence self-reported affective state” (Cuddy et al., 2018, p. 656–666).

Although many studies have looked at the different associations between emotion and movement (i.e., emotional expression through movement, emotion recognition from motor emotional expressions and emotion elicitation using movement), different studies used different movement analysis methods to describe those associations, causing a lack of a common base upon which it is convenient to compare results from different studies. A couple of researchers chose to overcome this difficulty by creating a systematic movement analysis method: (Dael et al., 2012a, p. 97–121) investigated emotional movement through the Body Action and Posture coding system which they created, and (Huis in ‘t Veld and Van Boxtel, 2014a; Huis in 't Veld and Van Boxtel, 2014b, p. 249–264; p. 1–13) created the Body Action Coding System which looks at muscle activation patterns during the perception and expression of different emotions. However, in this study, we chose to continue our previous work (Shafir et al., 2016, p. 2030), and therefore used Laban Movement Analysis (LMA), which is an existing comprehensive movement analysis system. Our decision to use LMA derived from LMA's advantages as a tool for describing and analyzing movement. First, it has the benefit of being a single descriptive language that can be used for both research and therapy, as well as its usefulness as a cross-cultural and cross-disciplinary movement language: LMA is a well-established internationally recognized system for describing and understanding body movements (Amighi, 1999), which has been widely used by researchers from different fields such as animation (Chi et al., 2000, p. 173–182), robotics (Lourens et al., 2010, p. 1256–1265; Masuda et al., 2010, p. 372–380), affective computing and motion capture (Bernstein et al., 2015a,b, p. 1394–1398; p. 37–44), as well as by Dance Movement Therapists for describing and assessing their patient's emotional movement, for planning interventions and for discussing movement with clients (Tortora, 2011, p. 242–254). Second, the use of LMA in diverse research studies points to its comprehensiveness as a motor analysis method (for a more detailed review about the use of LMA in recent research see Shafir et al., 2016). Third, LMA's capacity for detailed notation of movement through symbols (called Motif writing) enables the study of an unlimited number of movements containing specific motor components, instead of using a limited number of pre-determined specific motor sequences. Fourth, using Motifs enables the study of clean movement data, uncontaminated by co-occurring movement components that might be unintentionally introduced through live or video demonstration. Lastly, several researchers who studied emotional movement were influenced by LMA, or investigated very similar characteristics to Laban's movement components (De Meijer, 1989, p. 247–268; Sawada et al., 2003, p. 697–708; Winters, 2008, p. 84–105), and others specifically used Laban terms as part of their research (Crane and Gross, 2013, p. 91–105; Shafir et al., 2016). Moreover, (Gross et al., 2012) found that using LMA enabled identification of more differences between emotions than using kinematic analysis.

LMA describes movement through four main movement categories: Body, Space, Shape and Effort. The Body category describes “what is moving,” e.g., which body parts are moving, and the coordination of these parts as well as basic actions such as walking or jumping. The Space category describes “where the body moves,” such as the direction of a movement (up or down, forward or backward or sideways or across), the planes the movement occurs in, as well as use of personal Kinesphere (Kinesphere is the sphere of space around the body in which our movement occurs, the space that we can access with our limbs without taking a step to a new place), paths in the general space, and more. The Shape category refers to changes in the shape of the body itself, as we move in relation to the surroundings, to others and to our needs. We observe Shape when we note such things as whether the body encloses or spreads, rises or sinks. The last movement category is Effort. Effort reflects the mover's inner attitude toward the movement. Effort can be manifested in four different Factors: Weight, Space, Time, and Flow, each spanning two poles. Weight-Effort spans between the poles of *Strong* and *Light* and refers to the amount of force invested in the movement. When giving in to gravity's pull without activation of Weight-Effort, the movement can be classified as Heavy/Passive-Weight or limp. Space-Effort ranges between *Direct* and *Indirect* and refers to the attitude toward the movement's direction. Time spans from *Sudden* to *Sustain*, and refers to the acceleration and deceleration of movement. Flow expresses the mover's attitude toward controlling the progression of movement, from a higher control–*Binding* to little control or moving with abandon–*Freeing* (Studd and Cox, 2013).

Using the knowledge that execution, imagination, and observation of emotional movements can enhance affect (Shafir et al., 2013, p. 219–227), Shafir et al. (2016) took this one step further and used LMA to identify which aspects of movement might be responsible for enhancing the specific emotions of happiness, sadness, fear, and anger. They coded validated (Atkinson et al., 2004, p. 717–746) video-clips of whole body emotional expressions to examine which Laban motor components appeared in those clips, and then asked LMA experts to move different combinations of those motor components and to note which emotion was enhanced by moving each combination [for further explanation about the methods used in that study see also (Tsachor and Shafir, 2019, p. 572)]. Statistical analysis of these data yielded the following results: Happiness was enhanced by the Laban motor components of: Jumping, Rhythmic (reinitiating) movements, Spreading, Free-Flow, Lightness, moving Up, and Rising. Sadness was enhanced by the Laban components: Passive-Weight, Arms touching the upper body, Sinking and dropping the head. The main Laban movement components enhancing fear were Retreating, Condensing, Bind-Flow, moving Backwards, and Enclosing. Anger was enhanced by the Laban components: Strong-Weight, Sudden-Time, Advancing, and Direct movements. Interestingly, their study's findings pinpoint components noted in most of the existing literature regarding the connection between specific movements and emotions, when “translating” those specific movements into Laban terms.

In this study we aimed to investigate whether those LMA components (found to elicit a certain emotion when moved) will also be recognized by observers as expressing that emotion, regardless of the mover's emotional intent when moving. This study tests the strength of- and expands the associations between Laban motor components and specific emotions found in Shafir et al. (2016), and aims to refine our understanding of how we perceive emotion from whole body movement. We hypothesized that the same motor components which elicited certain emotions when included in a movement, will cause that movement to be recognized as expressing that same specific, associated emotion, even when the mover does not intend to express an emotion.

A unique aspect of our research design should be noted: In all previous studies of emotion recognition from bodily expressions, the stimuli were video clips of movers who intended to express a certain emotion through whole body movement. In this study, movers in the stimuli were not asked to express emotion, but rather instructed to move with specific motor characteristics. Therefore, “emotion attribution” is the most accurate term for what participants perceived and named when observing movement clips in this study (as opposed to “emotion recognition”), because one cannot recognize an emotion which was not expressed. Nevertheless, since our basic assumption is that both “recognition” and “attribution” are based on the same internal association between a certain set of movement characteristics and a specific emotion, and because it is this internal association that we investigate and try to characterize in this study, we decided to use in this paper the term “emotion recognition” and not “emotion attribution,” in order not to confuse the reader with a new term.

## Materials and Methods

### Participants

Sixty-two healthy males (*n* = 31) and females (*n* = 31), age 19–48 years old (Mean = 32.5, *SD* = 8.8) participated in the study. Participants were from diverse personal, ethnic backgrounds: 75.8% of the participants were identified ethnically as Jewish, 6.5% as Muslim, 6.5% as Druze, 4.8% as Christian and 6.5% defined themselves as having other religion. Exclusion criteria were: chronic or psychiatric illnesses, any movement disability, and taking psychiatric medication. All participants joined voluntarily and signed a written informed consent. The study was approved by the ethical committee of the Faculty of Social Welfare and Health Sciences, University of Haifa.

### Stimuli

For each set of LMA components found by Shafir et al. (2016) to elicit a specific emotion when moved, we created video stimuli based upon all possible combinations of two, three, and four components from that set. Overall, 59 combinations were created and recorded: 11 for each of the emotions: sadness, anger and fear (which had four components in each set), 20 for happiness (which had six components in its set) and six for neutral (i.e., no specific emotion). Anger combinations were composed of the components: Strong, Sudden, Advance, and Direct. Sadness from the components: Passive-Weight, Arms-to-upper-body, Sink, and Head-drop. Fear combinations were composed of: Retreat, Bind, Condense and Enclose, and Twist and Back. Happiness combinations included: Jump, Rhythmicity, Spread, Free and Light, Up and Rise, and Rotation. Although Shafir et al. (2016) did not find rotation as a component enhancing happiness, this component was added following the results of an additional emotional-movements study of ours. The neutral combinations were composed of LMA components that were not associated with any of the other emotions, such as Indirect Space Effort or Sustained Time Effort. When creating the fear and happiness combinations, some motor components were combined together due to their resemblance, or because they often appear together, to reduce the number of possible combinations for each emotion. These were: “Condense and Enclose” and “Twist and Back” for fear, and “Free and Light” and “Up and Rise” for happiness. More combinations were created for happiness because happiness had more associated LMA components.

Five Certified Laban Movement Analysts (CMAs) were filmed performing short improvised (unscripted) movement sequences for each combination, composed of all the components included in that combination, and only those components. The CMAs were instructed to move those components in any way they chose to move, and emotional expression was not mentioned at all.

In order to verify that the components in the clips are the intended ones, and because it is very difficult to produce movements composed of only a few specific movement components, we asked four other CMAs to observe the videos, tag all dominant components and trim the video-clips into 3 s clips showing movements comprised predominately by the required components. To assure the reliability of the tagging and trimming clips procedure, we asked these four tagging CMAs (who were different people from those who moved the combinations of motor components) to separately tag a set of 20 clips (four for each of the emotions: anger, sadness, and fear, six for happiness and two neutral). Coders were asked to write which of the target components they recognized to be dominant in the clip and whether there were other dominant components, which were not meant to be analyzed. Fleiss (1971, p. 378) category-wise Kappa was computed as an index of inter-rater agreement between these four raters on categorical data, using the “irr” package of the R Foundation for Statistical Computing version (3.0.1). Results indicated very high inter-rater agreement reliability (kappa = 0.676, *z* = 56.3, *p* < 0.001), which allowed them to each code and cut part of the 113 clips independently from one another.

**Figure 1:**
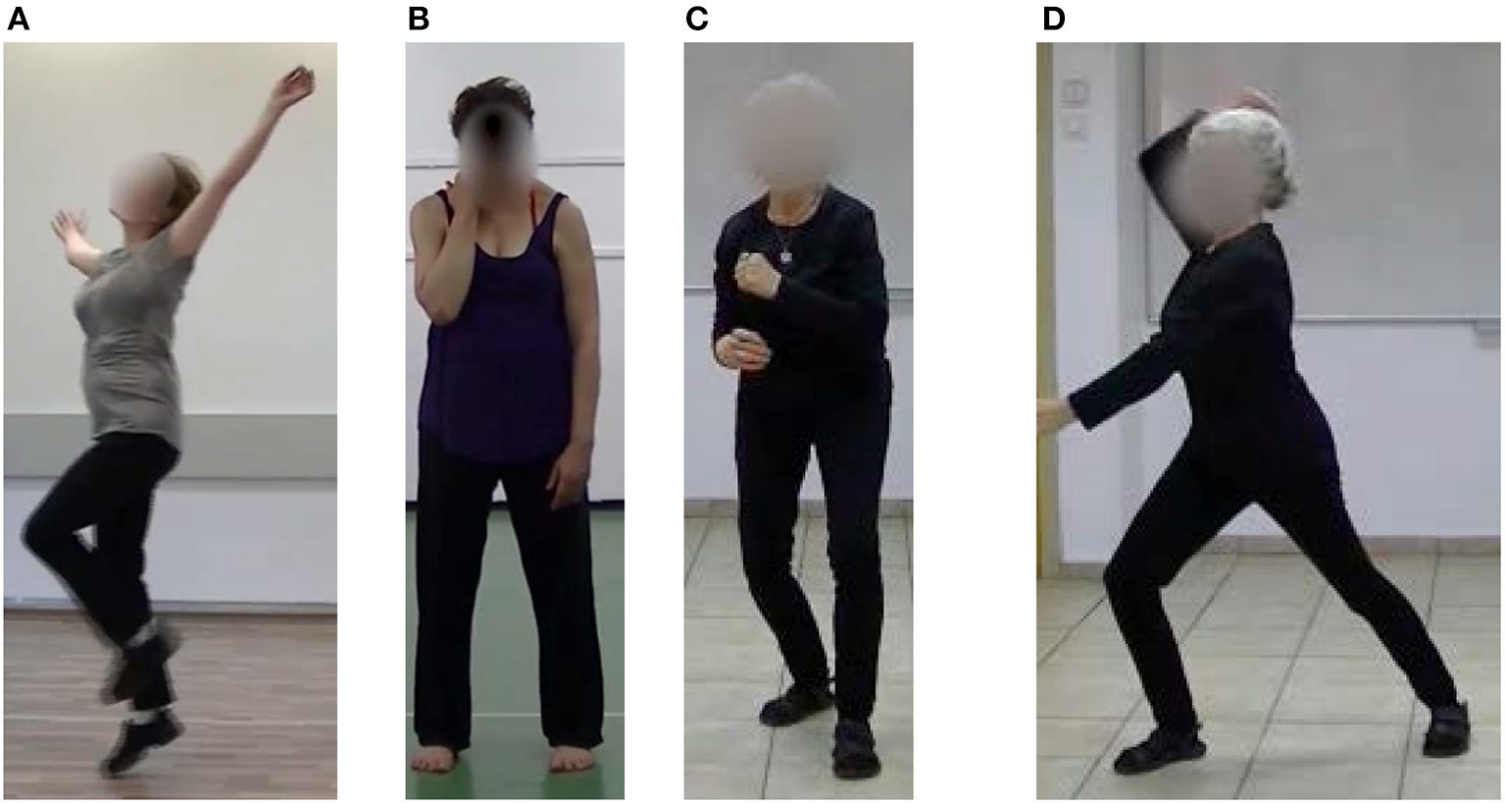
This figure shows a still shot from our stimuli clips of each of the studied emotions. **(A)** An image capturing a happy movement. The happiness components included Jump, Rhythmicity, Spread, Free and Light, Up and Rise, and Rotation. **(B)** An image capturing a sad movement. The sadness components included Passive Weight, Head drop, Arms to upper body, and Sink. **(C)** An image capturing a fear movement. The fear components included Bind, Retreat, Condense and Enclose, and Twist and Back. **(D)** An image capturing an angry movement. The anger components included Strong, Sudden, Advance, and Direct.

During the tagging procedure, in the case of the combined components (in happiness and fear), the pairs were considered present whenever one of the paired components or both were present in the movement. Out of the five clips produced for each components-combination (by five CMAs, one clip each), we extracted two in which (1) All intended LMA components predominated movement in the clip and (2) No unintended meaningful components were added (i.e., the movement did not include any component that was found by Shafir et al. (2016) to be associated with a different emotion). Although we aimed to select two clips for each combination, for one sadness combination, one fear combination and one happiness combination, only one clip met the criteria, and for one neutral combination we could not find any clip to withstand the criteria. This happened because three combinations generated by theoretical methodological considerations (to investigate *every* possible combination of all components) were in fact very difficult to perform motorically. For example: one theoretical sadness combination was composed of the components Arms to upper body and Sink. Most movers could not isolate only those two elements; some movers crossed arms in front of their upper body and went spatially down, legs bending, without sinking. Others succeeded in sinking, but to do so, they ended up adding in Passive Weight or a drop of the head. Thus, we ended up using 113 clips in this study: 22 for anger, 21 for each sadness and fear, 39 for happiness, and 10 for neutral. We then blurred the faces of the movers in all chosen clips to ensure that the emotion recognized in those clips was based only on bodily movement cues and not facial ones, and asked the participants to observe those clips and rate (forced choice) which emotion is expressed in each clip (see Figure 1).

Participants were asked to observe and rate the recognized emotion from 50 additional clips of bodily emotional expressions (10 for each of the emotions: sadness, happiness, anger, fear, and neutral emotion) from the validated set of Atkinson et al. (2004, p. 717–746). These validated clips were used for verifying participants' ability to recognize emotions from movement (to ensure that if participants didn't recognize emotions from our clips, it is because of the content of our clips and not because they have a problem with emotion recognition), and to compare participants' accuracy in emotion recognition from Atkinson's validated emotional bodily expressions to their accuracy in attributing the correct associated emotion to the combinations of Laban motor components. One clip portraying disgust from Atkinson et al. (2004, p. 717–746) set (an emotion that exists in their set but was not included in the current study) was mistakenly included in our study as a happiness clip, and results pertained to recognition of that clip were therefore omitted from analysis.

### Questionnaire

A demographic questionnaire collected data regarding age, gender, place of birth, family status, current occupation, ethnicity, and education.

### Procedure

Meetings with the participants took place in a quiet room at their convenience. After signing a consent form, participants were asked to watch the stimuli clips which were displayed to them using a 15.6″ laptop computer. All clips were presented using E-prime software (www.pstnet.com/eprime.cfm). Following the presentation of each clip, participants were asked to identify the expressed emotion (forced choice). The participants were encouraged to reply their most immediate answer. The presentation was divided into four parts: First there was a short training in which participants were asked to observe and respond to four of the LMA-component clips. After this training, participants were encouraged to ask questions about the procedure. Then, the first block of the LMA clips that included 57 clips was presented. At the end of the first block participants were invited to take a short refreshment break of up to 5 min, and then were presented the second block that included 56 clips. After another short refreshment break, participants were asked to watch and respond to the 50 clips of Atkinson's bodily emotional expressions. Each of the first two blocks had a similar number of clips from each emotion. Within each block, the clips were presented in a random order, but the blocks were presented in the same order for all participants. Lastly, participants were asked to fill in the demographic questionnaire. At the end of the session, an explanation regarding the study was given to participants who were interested in it.

### Statistical Analysis

#### Emotion Recognition From Movement

The percentage of correct recognition (attribution) was defined as the percentage of “correctly” (match-expectancy) recognized clips from all clips presented. Emotion recognition was considered correct if the emotion recognized matched the emotion associated with the movement components presented, based on Shafir et al. (2016). Percent correct recognition was calculated for each emotion and for the entire sample, separately for the Laban clips and the (Atkinson et al., 2004, p. 717–746) ones. To ensure that movements were recognized above chance level, we calculated the probability to have correct recognition by chance in n and more observations, using Bayes' theorem (Johnson and Bhattacharyya, 2010). We then looked for the threshold above which the probability to have that emotion recognition level is ≤0.05. The thresholds that were found based on this test were: 21.8% for the clips associated with anger, sadness and fear (i.e., any recognition level equal to, or higher than 21.8% was statistically significant as expressing above chance recognition), 21.3% for the clips associated with happiness, 22.9% for the clips associated with neutral and 20.7% for the entire sample.

Taking into consideration that participants' choices of emotions may be inherently biased, we also used Wagner's (Wagner, 1993, p. 3–28) method for unbiased hit rate and chance proportions. According to this method we calculated the arcsine transformed unbiased hit rate accuracy scores per participant for each emotion separately. We then computed and arcsine transformed the chance proportion scores per participant for each emotion as well. Finally, we conducted pairwise comparison with Bonferroni correction between the arcsine transformed unbiased hit rates and the arcsine transformed chance proportion for each emotion (happy, sad, fear, and anger) separately. Because we repeated the pairwise comparison with four emotions, the Bonferoni correction adjusted the threshold for significance to 0.05/4 = 0.013.

#### Exploring the Contribution of Specific Movement Components to Emotion Recognition

After establishing that our clips were recognized as expressing the emotions associated with the movement components comprising those clips, we wanted to investigate which components contributed most to the recognition level of their associated emotion. To do that, for each emotion (happiness, sadness, fear, and anger), a separate logistic regression model was fitted to predict the recognition of that emotion. We organized our data so that for each case (i.e., each clip associated with a specific emotion) the independent binary variables in this logistic regression were the presence or absence of each LMA component associated with that emotion as predictors, and the dependent variable was the number of expected recognitions (“event”) vs. the number of recognitions other than the expected one (“non-event”). Thus, the binary variables of the presence vs. absence of the LMA components: Jump, Rhythmicity, Spread, Free and Light, Up and Rise, and Rotation were tested for the prediction of recognizing happiness. The binary variables of the presence vs. absence of the LMA components: Passive Weight, Arms-to-upper-body, Sink and Head-drop were tested for the prediction of recognizing sadness. The binary variables of the presence vs. absence of the LMA components: Retreat, Condense and Enclose, Bind, and Twist and Back were tested for the prediction of recognizing fear. Lastly, the binary variables of the presence vs. absence of the LMA components: Strong, Sudden, Direct, and Advance were tested for the prediction of recognizing anger.

Since anger was mainly confused with fear (i.e., most of the “anger clips” which were not recognized as expressing anger were recognized as expressing fear) and fear was mainly confused with sadness and anger (i.e., most of the “fear clips” which were not recognized as expressing fear were recognized as expressing sadness or anger), and since anger and fear were the least well-recognized emotions, we wanted to check if any of the components associated with anger and fear contributed to their “wrong” recognition, i.e., to the recognition of an emotion to which they were not originally associated with based on Shafir et al. (2016). To answer this question, another logistic regression model was fitted to predict the “wrong” recognition of fear by anger components, i.e., the binary variables of the presence vs. absence of the LMA components: Strong, Sudden, Direct, and Advance, which were originally associated with anger, were tested for the prediction of recognizing fear. Additional regressions were fitted to predict the “wrong” recognition of sadness and anger by fear components: The binary variables of the presence vs. absence of the LMA components: Retreat, Condense and Enclose, Bind, and Twist and Back were tested separately for the prediction of recognizing anger and sadness.

No interaction between predictors were tested in any of the regression models. All the regressions were calculated by SAS 9.4 program.

#### Comparison to a Validated Set of Emotionally Expressive Video Clips

To compare emotion recognition from Atkinson et al., 2004 validated clips to the emotion recognition from our clips (which were based on combinations of Laban components), all together and separately for each emotion, a two-way, five Emotion [anger, fear, sadness, happiness, and neutral] × 2 Clip-type [Atkinson, Laban] mixed-model repeated-measures analysis of variance (ANOVA) was run. When interactions were established, paired *t*-tests were used to assess the difference between individual means. Studentized Maximum Modulus (SMM) corrections were implemented to account for multiple comparisons in these *post-hoc* tests.

## Results

### Emotion Recognition Accuracy

**Figure 2:**
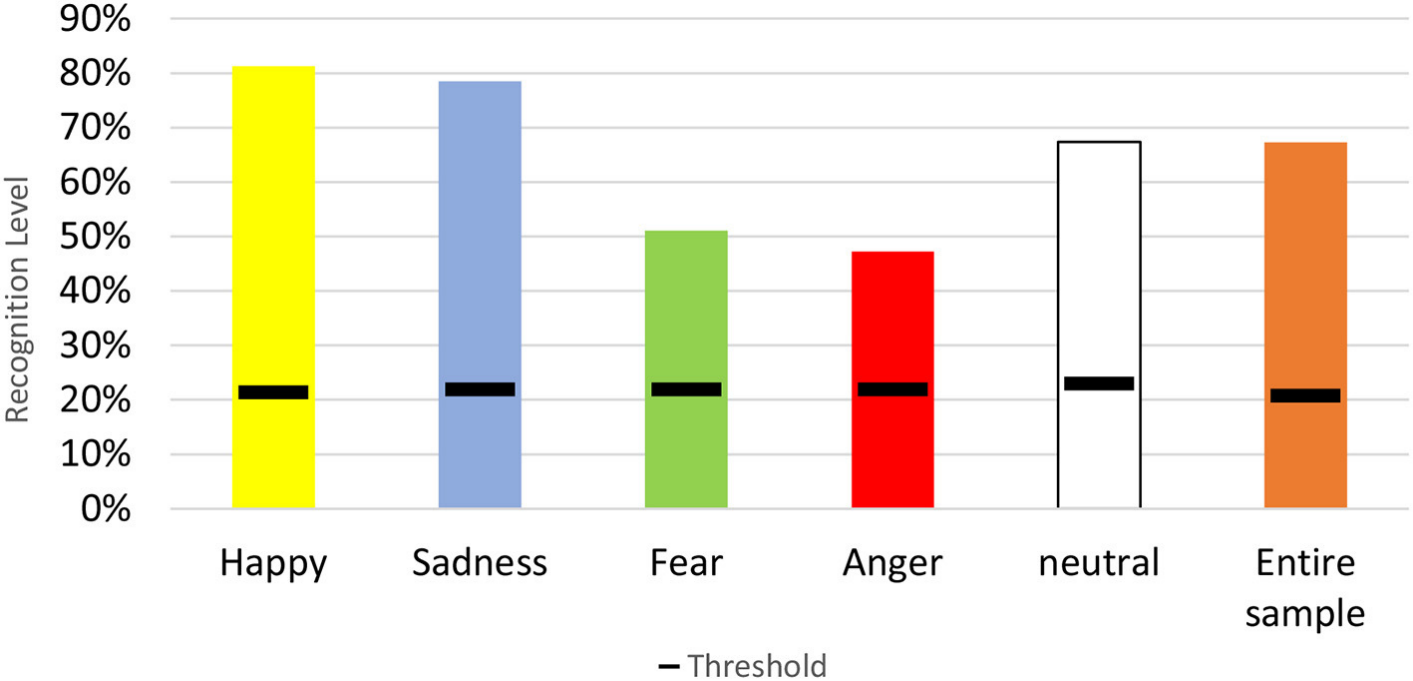
This graph shows the percent correct recognitions of the expected emotion from Laban clips with the threshold for random recognition level. Each emotion is represented by a different color: Yellow for happiness, blue for sadness, green for fear, red for anger and white for the neutral emotion. The entire sample is marked orange.

67.3% of all Laban clips were accurately recognized. Looking at each emotion separately, happiness was best recognized, with 81.3% correct recognition. Second was sadness with 78.5% of sadness-component clips recognized as expressing sadness. Then neutral (67.4%), fear (51.1%), and lastly anger in which 47.2% of the clips composed of anger-associated motor components were recognized by participants as expressing anger. Mean recognition levels for all emotions were much above the threshold for recognition above chance (Table 1 and Figure 2).

**Table 1:** This table shows the Laban error distribution.

	**Recognized emotion**				
**Intended emotion**	**Happy**	**Sadness**	**Fear**	**Anger**	**Neutral**
Happy	81.39	1.36	1.65	2.07	13.52
Sadness	0.61	78.57	5.07	5.38	10.37
Fear	0.61	23.12	51.15	9.14	15.98
Anger	2.57	3.67	26.17	47.29	20.31
Neutral	11.77	11.45	5.00	4.35	67.42

*All numbers represent percentages. Rows represent the intended (“correct”) emotions and columns represent the emotion that was recognized. Thus, the number at each cell represents the percentage of clips recognized as the column emotion from all clips associated with the row emotion. For example: 47.29% of all anger associated clips were recognized as anger and 26.17% of all anger clips were recognized as fear*.

As seen in the error distribution table (Table 1), while happiness and sadness (which had the highest recognition levels) were rarely confused with other emotions, anger clips were mostly confused with fear (26.17% of the Laban anger clips were mistakenly recognized as fear) and fear was often confused with sadness and to a lesser degree with anger (23.12 and 9.14% of fear clips were recognized as sadness and anger, respectively). All emotions were relatively highly confused with neutral: 20.31, 10.37, 15.98, and 13.52% of the anger, sadness, fear, and happiness clips, respectively, were recognized as neutral. Only 30% of the clips which were recognized as neutral were actually made of the neutral components, indicating that participants tended to choose neutral when the emotion was not clear enough for them, therefore, when they chose an emotion, they were likely to think they recognized it well.

**Figure 3:**
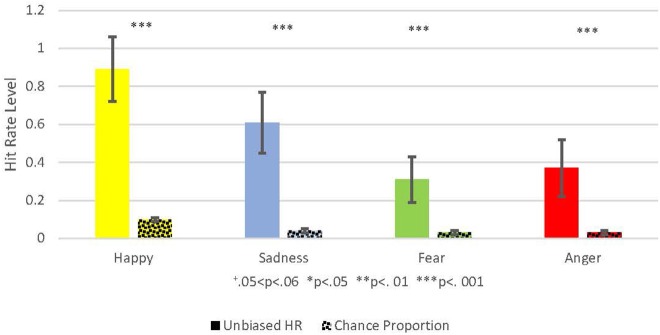
This figure shows the Comparison between the Unbiased Hit Rate and the Chance Proportion, i.e., the hit rate that would have been expected by chance. Unbiased Hit Rate is colored with a full color, and the chance proportion is marked with dots. Each emotion is represented by a different color: Yellow for happiness, blue for sadness, green for fear, red for anger. Hit Rate mean is represented by the bar's height and standard deviation by the black brackets. The Significance level is marked: ^+^0.05 < *p* < 0.06, *p < 0.05, **p < 0.01, ***p < 0.001.

An additional analysis, which compared the unbiased hit rates with the chance proportions, also yielded similar results (Figure 3): the pairwise comparison (2-tailed) between happiness unbiased hit rate (*M* = 0.89, *SD* = 0.17) and the happiness chance proportion (*M* = 0.1, *SD* = 0.01) indicated a significant difference (*t* = 38.32, *p* < 0.001). The pairwise comparison (2-tailed) between sad unbiased hit rate (*M* = 0.61, *SD* = 0.16) and the sad chance proportion (*M* = 0.04, *SD* = 0.01) also indicated a significant difference (*t* = 26.67, *p* < 0.001). The pairwise comparison (2-tailed) between fear unbiased hit rate (*M* = 0.31, *SD* = 0.12) and the fear chance proportion (*M* = 0.03, *SD* = 0.01) indicated a significant difference (*t* = 18.84, *p* < 0.001). Lastly, the pairwise comparison (2-tailed) between anger unbiased hit rate (*M* = 0.37, *SD* = 0.15) and the anger chance proportion (*M* = 0.03, *SD* = 0.01), indicated a significant difference (*t* = 18.61, *p* < 0.001).

### Results for the Exploratory Components Analysis

Fifteen out of 18 movement components significantly increased the likelihood that a clip will be recognized as the expected emotion, one component decreased the likelihood that a clip which contains it will be recognized as the expected emotion and two components out of the 18 tested increased the likelihood that a clip which contains them will be recognized as expressing an emotion different from the expected emotion (Table 2).

**Table 2:** This table describes movement components ability to predict the recognition of their associated emotions.

**Emotion**	**Component**	**Estimate**	**SE**	**Wald S**.	**OR**	**Lower CL**	**Upper CL**
**Happy**	**Jump**	**2.73**	**1.02**	7.20^**^	**15.29**	**2.09**	**112.09**
**Happy**	**Rhythmicity**	**3.54**	**1.02**	11.95^***^	**34.64**	**4.64**	**258.47**
**Happy**	**Spread**	**3.15**	**1.01**	9.63^**^	**23.42**	**3.20**	**171.51**
**Happy**	**Free and light**	**2.74**	**1.02**	7.24^**^	**15.48**	**2.11**	**113.81**
**Happy**	**Up and rise**	**2.39**	**1.03**	5.43^*^	**10.94**	**1.46**	**81.89**
**Happy**	**Rotation**	**2.54**	**1.02**	6.24^*^	**12.68**	**1.73**	**92.98**
**Sad**	**Passive Weight**	**0.49**	**0.17**	8.37^**^	**1.64**	**1.17**	**2.28**
**Sad**	**Arms to upper body**	**0.18**	**0.17**	**1.08**	**1.19**	**0.85**	**1.68**
**Sad**	**Sink**	**0.90**	**0.18**	23.92^***^	**2.47**	**1.72**	**3.55**
**Sad**	**Head-drop**	**2.03**	**0.18**	128.48^***^	**7.60**	**5.35**	**10.79**
**Fear**	**Retreat**	**0.98**	**0.13**	53.32^***^	**2.65**	**2.04**	**3.45**
**Fear**	**Condense and enclose**	**−0.26**	**0.13**	3.92*	**0.77**	**0.60**	**0.99**
**Fear**	**Bind**	**0.03**	**0.13**	**0.04**	**1.03**	**0.79**	**1.33**
**Fear**	**Twist and back**	**1.12**	**0.13**	69.67^***^	**3.06**	**2.35**	**3.98**
**Anger**	**Strong**	**1.32**	**0.15**	80.22^***^	**3.74**	**2.80**	**4.99**
**Anger**	**Sudden**	**2.24**	**0.16**	205.78^***^	**9.42**	**6.93**	**12.80**
**Anger**	**Advance**	**1.16**	**0.14**	66.15^***^	**3.20**	**2.42**	**4.23**
**Anger**	**Direct**	**0.44**	**0.13**	11.58^***^	**1.56**	**1.21**	**2.02**

Each emotion is colored with a different color: happiness is yellow, sadness is blue, fear is green, and anger is red. As can be seen from the table most components significantly increased the recognition of their associated emotion, and one component significantly decreased the recognition of the associated emotion. SE, Standard error of the estimate; Wald S., Wald Statistic; OR, Odds Ratio; Lower CI, Lower confidence interval; Upper CL, Upper confidence interval; Significance level was marked:

* p < 0.05,

** p < 0.01,

*** *p < 0.001*.

#### Happiness

All components associated with happiness (Jump, Rhythmicity, Spread, Free and Light, Up and Rise, and Rotation) significantly increased the likelihood of happiness-recognition when present in a movement. The components that increased the likelihood of happy recognition most were rhythmicity and spreading. Rhythmicity increased the likelihood of happy recognition by nearly 35 times (*OR* = 34.64, *p* < 0.001) and spreading by 23 times (*OR* = 23.416, *p* = 0.002). The components Free and Light and Jump increased expected recognition likelihood by 15 times (*OR* = 15.48, *p* = 0.007 and *OR* = 15.291, *p* = 0.007, respectively), Rotation by nearly 13 times (*OR* = 12.67*, p* = 0.013), and lastly, Up and Rise increased the likelihood of happy recognition by 11 times (*OR* = 10.94, *p* = 0.02).

#### Sadness

The LMA components that were tested for predicting the recognition of sadness were: Passive Weight, Arms-to-upper-body, Sink and Head-drop. Three of them significantly increased the likelihood of sadness recognition when present in a movement. The component that increased the likelihood of sadness recognition most was Head-drop, which increased the likelihood of sadness recognition by nearly eight times (*OR* = 7.601, *p* < 0.001). Sink increased expected recognition likelihood by more than two times (*OR* = 2.471*, p* < 0.001), and Passive Weight by nearly two times (*OR* = 1.636, *p* = 0.008). The presence of Arms-to-upper-body was not found to significantly increase the likelihood of sad recognition when present in a movement (*OR* = 1.197, *p* = 0.298).

#### Fear

The LMA components that were tested for predicting the recognition of fear were: Retreat, Condense and Enclose, Bind, and Twist and Back. Two of them significantly increased the likelihood of fear recognition when present in a movement. Twist and Back increased the likelihood of fear recognitions by three times (*OR* = 3.058, *p* < 0.001) and Retreat increased fear recognition by more than two times (*OR* = 2.654, *p* < 0.001). The presence of Bind was not found to significantly increase the likelihood of fear recognition when present in a movement (*OR* = 1.026, *p* = 0.843) and Condense and Enclose significantly decreased the likelihood of fear recognition by 0.77 times (*OR* = 0.773, *p* = 0.047).

The component Condense and Enclose was found to significantly increase the likelihood of sadness recognition by four times (*OR* = 4.145, *p* < 0.001), and the component Bind was found to significantly increase the likelihood of anger recognition by over three times (*OR* = 3.75, *p* < 0.001) when it was present in a movement. All other fear related components (Twist and back, and Retreat) were either negatively related or not related to recognition of sadness or anger (Table 3).

**Table 3:** This table describes the relation between the ability of the components of anger and fear (the emotion least well-recognized) to increase the likelihood for recognitions of the emotions which they were most confused with (anger components with fear, and fear components with sad and anger).

**Component**	**Expected emotion**	**Recognized emotion**	**Estimate**	**SE**	**Wald S**.	**OR**	**Lower CL**	**Upper CL**
**Strong**	**Anger**	**Fear**	−1.49	0.17	74.46^***^	0.23	0.16	0.32
**Sudden**	**Anger**	**Fear**	−1.49	0.17	74.46^***^	0.23	0.16	0.32
**Advance**	**Anger**	**Fear**	−1.56	0.17	84.72^***^	0.21	0.15	0.29
**Direct**	**Anger**	**Fear**	−0.69	0.16	17.84^***^	0.50	0.36	0.69
**Retreat**	**Fear**	**Sad**	−1.13	0.15	54.29^***^	0.32	0.24	0.44
**Condense and enclose**	**Fear**	**Sad**	**1.42**	**0.20**	49.73^***^	**4.14**	**2.79**	**6.15**
**Bind**	**Fear**	**Sad**	0.14	0.16	0.78	1.15	0.84	1.57
**Twist and back**	**Fear**	**Sad**	−0.67	0.15	18.65^***^	0.51	0.38	0.69
**Retreat**	**Fear**	**Anger**	0.35	0.23	2.36	1.42	0.91	2.21
**Condense and enclose**	**Fear**	**Anger**	0.39	0.23	2.91	1.48	0.94	2.34
**Bind**	**Fear**	**Anger**	**1.32**	**0.28**	21.37^***^	**3.75**	**2.14**	**6.57**
**Twist and back**	**Fear**	**Anger**	−1.20	0.22	30.04^***^	0.30	0.19	0.46

Components that predicted an unexpected emotion were colored with the color of that emotion: red for anger, green for fear and blue for sadness. For example: Condense and Enclose which was originally associated with fear (green), was found to increase the likelihood of recognizing sadness (blue). SE, Standard error of the estimate; Wald S., Wald Statistic; OR, Odds Ratio; Lower CI, Lower confidence interval; Upper CL, Upper confidence interval; Significance level was marked:

* p < 0.05,

** p < 0.01,

*** *p < 0.001*.

#### Anger

All anger components (Strong, Sudden, Direct, and Advance) significantly increased the likelihood of anger recognition when present in a movement. The component that increased the likelihood of anger recognition most was Sudden, which increased the likelihood of anger recognition by over nine times (*OR* = 9.42, *p* < 0.001). The components Strong and Advance have both increased expected recognition likelihood by more than three times (*OR* = 3.744, *p* < 0.001 and *OR* = 3.199, *p* < 0.001, respectively), and Direct increased the likelihood of anger recognition by 1.6 times (*OR* = 1.561, *p* < 0.001).

Although many of the clips of anger-associated components were unexpectedly recognized as expressing fear, none of the individual anger components was found significantly predicting fear recognition. Moreover, all anger components were found significantly negatively related to the unexpected recognition of fear: Advance decreases the likelihood of fear recognition in 79% (*OR* = 0.209, *p* < 0.001), Strong and Sudden decrease fear recognition in 77% (*OR* = 0.226, *p* < 0.001 for both), and Direct decreases fear recognition in 50% (*OR* = 0.502, *p* < 0.001).

#### Comparison to a Validated Set of Emotionally Expressive Video Clips

**Figure 4:**
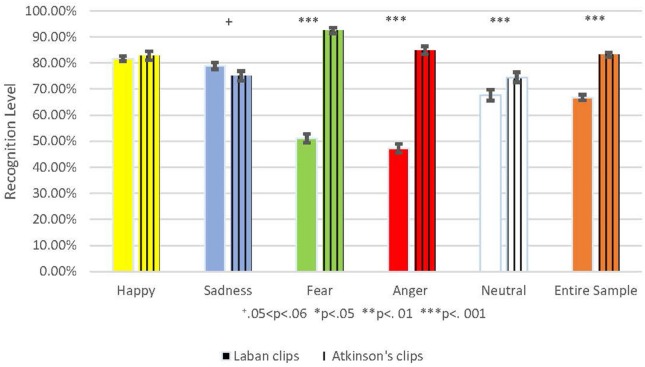
This shows the Comparison between the percent of correct emotion recognition from the Laban stimuli to those from Atkinson's validated clips. The Laban recognition level is colored with a full color, and Atkinsons' validated clips are marked with vertical lines. Each emotion is represented by a different color: Yellow for happiness, blue for sadness, green for fear, red for anger, and white for the neutral emotion. The entire sample is marked orange. Accuracy mean is represented by the bar's height and standard deviation by the black brackets. The significance level is marked: ^+^0.05 < *p* < 0.06, *p < 0.05, **p < 0.01, ***p < 0.001.

The recognition results for Atkinson's (Atkinson et al., 2004, p. 717–746) validated set showed high recognition levels for these clips (81.5% of all clips were accurately recognized) indicating that the participants had good capability for emotion recognition from bodily expressions. Although our LMA-component stimuli were well-recognized, Atkinson's (Atkinson et al., 2004, p. 717–746) validated set was significantly better recognized for the entire sample: *F*_(4, 610)_ = 75.28, *p* < 0.001, as well as for some of the emotions: anger: *F*_(1, 610)_ = 217.52, *p* < 0.001, fear *F*_(1, 610)_ = 234.77, *p* < 0.001 and neutral *F*_(1, 610)_ = 6.97, *p* = 0.008. No difference was found for happiness *F*_(1, 610)_ = 0.34, *p* = 0.562. Moreover, sadness was recognized slightly better in the Laban set, with close to statistical significance *F*_(1, 610)_ = 3.68, *p* = 0.056 (Figure 4).

## Discussion

Consistent with our hypothesis, the results indicated that movements composed of motor components associated with specific emotions were recognized as expressing those emotions, even when the mover did not intend to express emotion. These findings complement Shafir et al. (2016) findings by showing that for the most part, the same components which elicited specific emotions when moved, also led to recognition of those emotions when being observed.

Following the finding that mirror neurons are activated in a very similar way during motor execution and during motor observation, it has been suggested that our emotional perception from movement during movement observation is based on simulation by the mirror neurons of the brain activation that happens during motor execution of the same movements (Heberlein and Atkinson, 2009, p. 162–177; Shafir et al., 2013, p. 219–227). Based on Damasio's somatic markers hypothesis (Damasio, 1999; Damasio et al., 2000, p. 1049–1056), during motor execution, it is the specific proprioceptive and interoceptive feedback from the body that generates the associated emotion. During motor observation, Raos et al. (2007) found activation of the somatosensory cortex in monkeys (Raos et al., 2007, p. 12675–12683), and Gazzola and Keysers (2008) and Valchev et al. (2016) found somatosensory activation during motor observation in humans (Gazzola and Keysers, 2008, p. 1239–1255; Valchev et al., 2016, p. 1205–1217). Such somatosensory activation during motor observation supports the idea of simulation of the proprioceptive and interoceptive feedback from the body during motor observation of a certain movement, a simulation which might elicit the associated emotion, similar to its elicitation by real proprioceptive input during motor execution of the same movement. Based on this idea, if the proprioceptive input from execution of a certain motor component is associated in the brain with a specific emotion and thus elicits that emotion, the observation of this motor component should simulate the same somatosensory activation and thus elicit the same associated emotion. Shafir et al. (2016) have demonstrated that certain movement components, when executed, are capable of enhancing specific associated emotions. In this study we have shown that 16 out of the 18 motor components taken from Shafir et al. (2016) study enhanced or activated the associated emotion when they were just observed, even when the mover did not intend ahead of time to express emotion. These results are in line with Damasio's idea that there are associations in the brain between certain proprioceptive feedback from the body and specific emotions, and support the notion that the mirror neurons create simulation of that proprioceptive feedback, and that this simulated proprioception causes us to feel those emotions and consequently to recognize them.

### Happiness

The Laban components in the happiness clips were: Jumping, Rhythmic (reinitiating) movements, Spread, Free-Flow, Light-Weight, Up and Rise, and Rotation. Since happiness recognition levels were very high (81.3%), the happiness clips were not readily confused with any other emotion, and there was no statistically significant difference between recognition accuracy of the happiness Laban stimuli clips and the validated happiness clips of Atkinson et al. (2004, p. 717–746), it is evident that the set of components that was found here and in Shafir et al. (2016) as associated with the recognition of happy movements, is very well-defined, i.e., includes all or most of the Laban motor components that most people associate with happiness, and no components that most people associate with other emotions.

Several previous studies found results similar to ours: De Meijer (1989, p. 247–268) found that in movements that were recognized as expressing positive valence such as joy, the torso tended to stretch, which is similar to our finding of spreading as a component associated with happiness. Both Atkinson et al. (2004, p. 717–746) and De Meijer (1989, p. 247–268) found an association between arms lifting movements and happiness, similar to our finding of Up and Rise as happiness components. Dael et al. (2012b, p. 1085) found an association between happiness and “up down repetitive hand action,” where such hand action can be actually described as having the “Up and Rise” and “Rhythmicity” Laban components. Lastly, jumping movements were previously found by Atkinson et al. (2004, p. 717–746) to often occur when we are happy.

It should be noted that our analysis indicated that the appearance of either Rhythmicity or Spread contributed the most to the recognition of happiness in the movement. This result echoes (Camurri et al., 2003, p. 213–225) who listed movements reaching out of the body center (which can be the spreading in Laban terms) and dynamic tension in movements that can be equivalent to rhythmicity, as movements characterizing joy expression in a dance. Interestingly, many other studies have found happy movements to be fast (De Meijer, 1989, p. 247–268; Sawada et al., 2003, p. 697–708; Crane and Gross, 2007, p. 95–101; Roether et al., 2009, p. 15), while in the current study Sudden (accelerating) movements were successfully attributed to anger. This attribution will be further discussed in the anger discussion. This finding, however, may be explained by the fact that the quality of sudden in LMA refers to the urgency of the movement as expressed by acceleration, rather than its velocity (i.e., fast).

### Sadness

The Laban components included in the clips that were recognized as expressing sadness were: Passive-Weight, Arms-to-upper-body, Sink, and Head-drop. The clips composed of sadness components had also a high recognition rate (78.5%), which was higher even than sadness recognition from Atkinson et al. (2004, p. 717–746) validated set, and they were not readily confused with other emotions. This was an unexpected result, since movers in this study were instructed to move only specific movement components with no emotional intention, while the actors in Atkinson et al. (2004, p. 717–746) were specifically instructed to express sadness.

This result strengthens the association between the movement components used in our study and the emotion of sadness. The association between sadness and the movement component Head-drop, was previously reported in Atkinson et al. (2004, p. 717–746) and Crane and Gross (2007, p. 95–101). The association between sadness and Sinking movement had also been previously demonstrated by Wallbott (1998, p. 879–896), who associated sadness with collapse of the upper body, and by Michalak et al. (2009, p. 202–221) who noted that sadness was marked by more slumped posture. Although these previous studies did not use the same terminology, it can be assumed it referred to movements that include the Sinking component. Surprisingly, the movement component Arms to upper body, which was found related to sadness in previous studies (Atkinson et al., 2004, p. 717–746; Crane and Gross, 2007, p. 95–101) and was expected to be found related to sadness in the current study, was not found to increase the likelihood of recognizing a sad emotion from a movement.

It should be noted that previous studies have characterized sadness expressions also by Free Flow, Indirect focus, and Light weight (Crane and Gross, 2013, p. 91–105) as well as slow movements (De Meijer, 1989, p. 247–268; Atkinson et al., 2004, 717–746; Crane and Gross, 2007, p. 95–101; Roether et al., 2009, p. 15; Crane and Gross, 2013, 91–105). Interestingly, in this study, Free flow and Light weight were associated with the emotion of opposite valence: happiness. It is possible that this discrepancy was due to differences in the coding procedures, since Crane and Gross (2013) used novice, unprofessional coders, while in our study professional CMAs were coding. Although novice coders' observations may be closer to the everyday psychological processing of emotional movement, they may also be less precise than those by professional coders, who are equipped to see subtle differences, and hence can lead to slightly different movement interpretations.

### Fear

The components in the fear clips were: Retreat, Bind, Condense and Enclose, and Twist and Back. Although the recognition of fear level was higher than what would have been expected by chance, it was yet relatively low, and the fear clips were readily confused with the sadness and anger clips. The logistic regression model revealed that only the presence of the components Twist and back and Retreat significantly increased the likelihood of fear recognition. This result is similar to Dael et al. (2012b, p. 1085) finding that panic, fear, and anxiety were associated with “backward body lean.”

On the other hand, the component Condense and Enclose was found to significantly decrease the likelihood of fear recognition and increase the likelihood of sadness recognition. This finding is similar to De Meijer (1989, p. 247–268) finding that fear was easily confused with other negative emotions. It also corresponds with Roether's et al. (2009, p. 15) finding of the association between fear and sadness and “being small,” since Condense and Enclose indeed make the body smaller. Such an association also makes sense in evolutionary terms, by making oneself small and “unseen” when facing a threat or a predator. Since the autonomic nervous system may cause one of three movement patterns in response to threat (fight, flight, and freeze), it is possible that fear has also several movement patterns, which could sometimes overlap with other emotions. This could be so in particular with sadness, which, like fear, is associated with withdrawal (as opposed to approach), and which is defined by some researchers as derived from separation distress, i.e., the *fear* of being alone (Panksepp and Yovell, 2014, p. 383–393).

Unexpectedly, the component Bind was also not found to be related to recognition of fear, but increased the likelihood of anger recognition. This result will be discussed at the anger discussion paragraph.

### Anger

The LMA components that were found as contributing to anger recognition were: Strong, Sudden, Advance and Direct. Although only 47.2% of the clips that had anger components were recognized as expressing anger, anger recognition from Laban movement components was still above chance level, and the logistic regression model revealed that all anger components significantly increase the likelihood of anger recognition when present in a movement.

These results indicate that these four components: Strong, Sudden, Direct, and Advance) are probably crucial for anger recognition. Other studies have also found the same or similar components as expressing anger: Crane and Gross (Crane and Gross, 2013, p. 91–105) found that angry gait was associated with *direct, strong*, and *binding* qualities; Sawada et al. (2003, p. 697–708) found that movements expressing anger were *stronger* and *faster* than movements expressing sadness or happiness and Roether et al. (2009, p. 15) found that angry gait tended to be *fast* with large steps compared to neutral gait. Although Roether et al. (2009, p. 15) and Sawada et al. (2003, p. 697–708) did not use a strict LMA terminology, we suggest that the *fast* movements they observed might be similar to the Laban component of Sudden (accelerating), which was one of the components related to anger in the current study.

Another similar, yet not identical match can be seen between findings of some *forward* movement in the angry movements in Crane and Gross (2013, p. 91–105) and Winters (2008, p. 84–105), and the Advance component associated with anger in the current study. In LMA terminology, forward indicates the direction in the general space in which the movement is progressing, while advance is a term related to the shape of the body. Usually when we advance in our torso, we also move forward in space, and vice versa, which might have caused other studies to relate to forward as a motor characteristic of anger expression.

Lastly, one component that was found to be associated with anger in Crane and Gross (2013, p. 91–105) and Winters (2008, p. 84–105), is Bind, which was associated in our study with fear. Interestingly, fear in our study was the emotion most confused with anger, i.e., many of the anger clips were mistakenly recognized as fear. Moreover, although Bind was a movement component originally associated with fear, the logistic regression model testing unexpected recognition of anger from fear clips, revealed that the presence of Bind in a movement increased the likelihood of anger recognition. These results may indicate that Binding is more important for the recognition of anger than for the recognition of fear. One possible explanation to the recognition of movements that contain Bind as expressing anger is that it is very difficult to perform a Strong but not Sudden movement without Binding when the movement is done in free space (e.g., as an open kinetic chain) and not against an object (in a closed kinetic chain), i.e., when not pushing an object. Thus, people often combine Strong with Bind. Another explanation might be related to the social taboo on anger expression: While “Punch” (i.e., Strong, Direct, and Sudden movement) may clearly express anger, substituting Binding (restraint of movement) for Suddenness may be more familiar to participants who often observe social situations in which anger is restrained (bound) vs. expressed/acted upon in a punch-like movement.

It should be mentioned that although misidentified anger clips were mostly confused with fear, the logistic regression model testing unexpected recognition of fear from anger clips, revealed that all anger components were negatively related to fear recognition and decreased the likelihood of fear recognition. Thus, the reason for the high confusion rate of anger related movements with fear recognition should be further investigated.

### Limitations of the Study

Although the population of this study was relatively diverse, more careful analysis of cross-cultural influences were not performed as our sample size was too small for that. Furthermore, all movers in the stimuli clips were adult Caucasian females, which may influence the way participants recognized the emotions expressed in their movements.

Additional limitations derive from the fact that there might be more Laban components or considerations that affect emotion recognition and which we did not test in this study. Although the LMA component stimuli were well-recognized, (Atkinson et al., 2004) validated set was significantly better recognized for the emotions of fear and anger, leading to the question about whether our set of components has identified all the components necessary for accurate recognition of emotions, and if there is a significance to sequencing or phrasing of these components. The phrasing (order, accent, and load) of movement components was not investigated here: In improvising the stimuli movement, some movers performed all the components in that combination simultaneously (all components at once) and others sequentially (one or two components first, then the others). This may have affected the strength of emotional expression, as the phrasing of movement is often significant to expression, and important to consider in future studies.

Another possible limitation that warrants further investigation is whether the body area in which the movement takes place has any effect on emotion recognition: Movers in our study who generated the stimuli clips were not instructed which specific body parts to use when moving the LMA components. Thus, some movers demonstrated the components with their whole body, and others just with limbs gestures or in isolated parts of the body. Future research will have to examine the effects of such considerations on emotion recognition.

### Conclusion

We set out to establish whether emotions could be recognized from brief glimpses of movement components associated with those emotions, and next to identify which, if any, components were more significant to recognition of emotion than others. Results from our study strongly indicate that specific components of movement contribute to our recognition of bodily expression of emotions, even when there is no intent on the part of the mover to express an emotion. Observing momentary movement of these components alone, in the absence of facial cues, context, or intent of the mover to experience or express emotions was sufficient for participants to identify the associated emotion. These results constitute new and important demonstration of the hypothesized underlying brain mechanism for emotion perception from body actions. That these LMA components, moved in unscripted improvised movements, significantly concur with components identified in previous studies, lends strength to the conclusion that specific movement components and their proprioceptive feedback are indeed associated in the brain with each emotion, across cultures and studies.

Our results showed the strongest correlations between: spreading (or expanding) rhythmic movements and happiness, dropping the head with expressing sadness, moving or turning backwards with fear and strong, sudden and advancing movement with Anger. This study also teased out movement components which overlap two basic emotions and may contribute to embodied experience of emotional complexity and blended emotions. For example, Binding movements encouraged a confusion between fear and anger and Condensing and Enclosing movements enhanced a confusion between fear and sadness.

Additionally, our review of previous studies findings using Laban Movement Analysis terms, combined with the results of our study, helps to pinpoint components noted in much of the existing literature regarding the connection between specific movements and emotions. The use of LMA terms for these findings and the literature review can provide a common research language for comparing results across studies and translating them into clinical application.

Lastly, our high inter-rater agreement about the observation of movement using LMA validates the high reliability of LMA as a movement analysis system, when carried out by certified observers.

The knowledge gained from this study's findings is clinically applicable, as it may help dance-movement, drama and music therapists understand their clients' bodily expressions and emotional movements, it may help them consider what intervention approach would serve each client best in a given situation, and help design their intervention and guide their suggestions to the clients, in relations to their emotional motor expressions. Moreover, the scientific strengthening to these associations between LMA components and emotional states, may encourage therapists to use LMA terms and language as an integral part of their assessments and professional communication.

## Ethics Statement

The study was approved by the ethical committee of the Faculty of Social Welfare and Health Sciences, University of Haifa.

## Author Contributions

AM contributed to the design of the study, to stimuli preparation, data acquisition, statistical analysis, and results interpretation. She wrote the manuscript. TS conceived the study. She contributed to the design of the study, to stimuli preparation, data acquisition, statistical analysis, and to results interpretation. She revised the manuscript. RT contributed to the design of the study, to stimuli preparation, and to results interpretation. She revised the manuscript. All authors approve the manuscript and agree to be accountable for all aspects of the work.

### Conflict of Interest Statement

The authors declare that the research was conducted in the absence of any commercial or financial relationships that could be construed as a potential conflict of interest.
