# Corrigendum: How Do We Recognize Emotion From Movement? Specific Motor Components Contribute to the Recognition of Each Emotion

**DOI:** 10.3389/fpsyg.2020.00184

**Published:** 2020-02-18

**Authors:** Ayelet Melzer, Tal Shafir, Rachelle Palnick Tsachor

**Affiliations:** ^1^Faculty of Social Welfare and Health Sciences, The Graduate School of Creative Arts Therapies, University of Haifa, Haifa, Israel; ^2^The Emili Sagol Creative Arts Therapies Research Center, University of Haifa, Haifa, Israel; ^3^School of Theater and Music, University of Illinois at Chicago, Chicago, IL, United States

**Keywords:** emotion recognition, Laban movement analysis, motor, emotion, movement, bodily emotional expressions, dance-movement therapy

In the original article, there was a mistake in Figure 3 and Figure 4 as published. Figure 3 was mistakenly published attached to the figure legend of Figure 4, while Figure 4 was mistakenly put together with the figure legend for Figure 3. The corrected Figure 3 and Figure 4 appear below.

**Figure 3:**
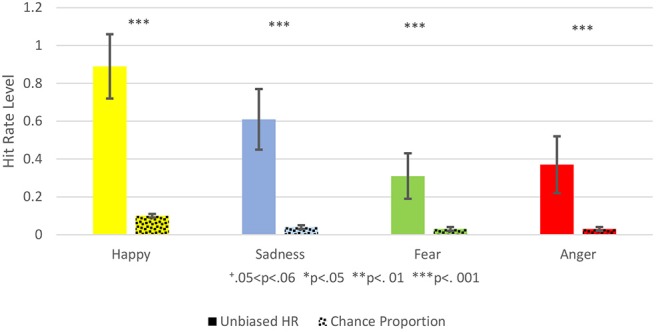
This figure shows the Comparison between the Unbiased Hit Rate and the Chance Proportion, i.e., the hit rate that would have been expected by chance. Unbiased Hit Rate is colored with a full color, and the chance proportion is marked with dots. Each emotion is represented by a different color: Yellow for happiness, blue for sadness, green for fear, red for anger. Hit Rate mean is represented by the bar's height and standard deviation by the black brackets. The Significance level is marked: ^+^0.05 < *p* < 0.06, *p < 0.05, **p < 0.01, ***p < 0.001.

**Figure 4:**
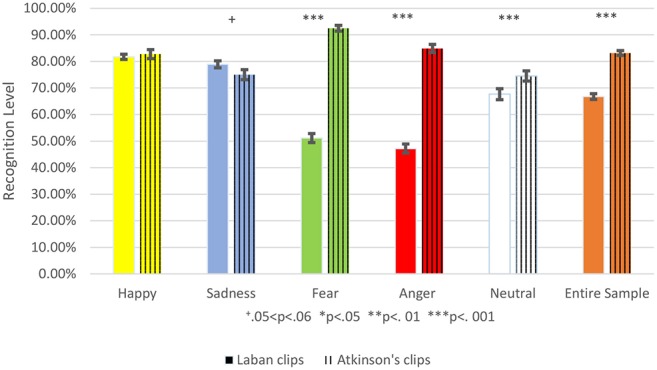
This shows the Comparison between the percent of correct emotion recognition from the Laban stimuli to those from Atkinson's validated clips. The Laban recognition level is colored with a full color, and Atkinsons' validated clips are marked with vertical lines. Each emotion is represented by a different color: Yellow for happiness, blue for sadness, green for fear, red for anger, and white for the neutral emotion. The entire sample is marked orange. Accuracy mean is represented by the bar's height and standard deviation by the black brackets. The significance level is marked: ^+^0.05 < *p* < 0.06, *p < 0.05, **p < 0.01, ***p < 0.001.

The authors apologize for this error and state that this does not change the scientific conclusions of the article in any way. The original article has been updated.

